# Chromogenic detection of yam mosaic virus by closed-tube reverse transcription loop-mediated isothermal amplification (CT-RT-LAMP)

**DOI:** 10.1007/s00705-018-3706-0

**Published:** 2018-01-08

**Authors:** Chukwuemeka K. Nkere, Joshua O. Oyekanmi, Gonçalo Silva, Moritz Bömer, Gabriel I. Atiri, Joseph Onyeka, Norbert G. Maroya, Susan E. Seal, P. Lava Kumar

**Affiliations:** 1 International Institute of Tropical Agriculture (IITA), Oyo Road, PMB 5320, Ibadan, Nigeria; 2 Department of Crop Protection and Environmental Biology, University of Ibadan, Ibadan, Nigeria; 3 National Root Crops Research Institute, Umudike, Nigeria; 4 Natural Resources Institute, University of Greenwich, Chatham Maritime, Kent, ME4 4TB UK

## Abstract

A closed-tube reverse transcription loop-mediated isothermal amplification (CT-RT-LAMP) assay was developed for the detection of yam mosaic virus (YMV, genus *Potyvirus*) infecting yam (*Dioscorea* spp.). The assay uses a set of six oligonucleotide primers targeting the YMV coat protein region, and the amplification products in YMV-positive samples are visualized by chromogenic detection with SYBR Green I dye. The CT-RT-LAMP assay detected YMV in leaf and tuber tissues of infected plants. The assay is 100 times more sensitive in detecting YMV than standard RT-PCR, while maintaining the same specificity.

**Electronic supplementary material:**

The online version of this article (10.1007/s00705-018-3706-0) contains supplementary material, which is available to authorized users.

Yam (*Dioscorea* spp.) is a popular vegetatively propagated staple crop cultivated for its starchy tubers in West Africa. Yam mosaic virus (YMV, genus *Potyvirus,* family *Potyviridae*) causes “mosaic disease”, the most economically important viral disease of yams, which can reduce yields by up to 40% [6, 17]. YMV is endemic in the West African “yam belt” stretching from western Cameroon to Côte d’Ivoire, which is known to be the largest edible-yam production region in the world [2]. YMV is most frequently detected in *D. rotundata,* the dominant yam species cultivated in the yam belt [2, 4, 6]. Since yams are clonally propagated using whole tubers, portions of the tubers (setts), or vine cuttings, the virus perpetuates from season to season and spreads to new areas along with the infected planting material [6].

The use of virus-free planting materials remains the most effective method to control the spread of YMV [8]. The development of sensitive, low-cost, field-based diagnostic tools is an important requirement to achieve this goal. Two common methods employed in the detection of yam viruses include enzyme-linked immunosorbent assay (ELISA) and reverse transcription polymerase chain reaction (RT-PCR), with the latter being the most widely used for YMV diagnosis [10, 13]. However, both ELISA and RT-PCR are lab based and require sophisticated equipment, precluding adoption of diagnostics in poorly equipped laboratories. Isothermal amplification methods, such as recombinase polymerase amplification (RPA) [13] and reverse transcription (RT) loop-mediated isothermal amplification (LAMP) [10], are relatively simple and rapid to perform, with high specificity and sensitivity, and are suitable for poorly equipped laboratories and on-site diagnosis [1, 9].

LAMP is a relatively simple method for specific amplification by strand displacement of DNA targets using *Bacillus stearothermophilus* (*Bst*) DNA polymerase under isothermal conditions in the range of 65 °C [9, 12]. Since the LAMP method uses four to six primers to recognize six to eight distinct regions on the template DNA, it has a higher specificity and amplification efficiency than other DNA amplification methods [10]. The LAMP assay has been shown to be as sensitive as or more sensitive than probe-based real-time PCR assays [14], and it has been successfully used for the detection of several plant viruses [5, 7, 11, 12]. In this study, we describe the development of a closed-tube (CT)-RT-LAMP assay as a simple and low-cost alternative for the detection of YMV-infected plants and seed yam tubers.

Yam (*D. rotundata*) leaf tissues and tubers were sourced from plants maintained in the screenhouse and experimental fields of the International Institute of Tropical Agriculture (IITA, Ibadan, Nigeria). About 100 mg of leaf sample was processed for total RNA extraction using a modified CTAB method [15]. Tuber tissues were excised by inserting a steel cork borer of 1 cm diameter into the tuber surface to a depth of about 1 cm, and total RNA was extracted from the plug of tissue. Extracted RNA was suspended in 50 µl of RNase-free water and stored at −20 °C until use. The RNA concentration was measured using a NanoDrop 2000 spectrometer (Thermo Scientific, UK) as per the manufacturer’s instructions. The total RNA concentration was adjusted to 100 ng/µl, and 2 µl was used as a template for all assays in this study, unless specified otherwise.

YMV infection in leaf and tuber samples used for the standardization of RT-LAMP was confirmed by RT-PCR using the primer pair YMV-F3x (5’GACAATGATGGACGGTGC3’) and YMV-B3x (5’ CTTTGCCATCAAATCCAAAC3’), which amplify a 320-bp region corresponding to the coat protein (CP) gene. The RT-PCR amplifications were set up in 12.5-µl reaction mixtures containing 2 µl of RNA template (at 100 ng/µl), 0.2 µM each primer, 0.2 mM each dNTP, 1 U of GoTaq DNA Polymerase (Promega, USA), 12 U of M-MLV reverse transcriptase (Promega, USA), and 1x GoTaq Reaction Buffer containing 1.5 mM MgCl_2_. The thermal cycle conditions of the RT-PCR consisted of an RT step at 42 °C for 30 min, followed by 95 °C for 5 min and 35 cycles of 95 °C for 30 s, 55 °C for 30 s, and 72 °C for 60 s, and a final extension of 72 °C for 10 min. RT-PCR products were analyzed by electrophoresis in a 1.2% agarose gel in tris-acetate-EDTA (TAE) buffer (pH 8.3) containing 0.05 µl of EZ-Vision Blue Light DNA Dye (Amresco, USA) per 1 ml. The agarose gel was visualized under UV light using a Gel Doc EZ Imager (Bio-Rad, France).

A set of six primers (Table 1, Supplementary Fig. 1) was designed to target the YMV coat protein (CP) using multiple sequence alignments of 40 YMV full CP nucleotide sequences available in the NCBI GenBank database using the Primer Explorer V4 software (http://primerexplorer.jp/e/). The final optimized RT-LAMP reaction conditions consisted of 13-μl reactions including 2 μl of RNA template, 0.16 μM each of primers YMV-F3 and YMV-B3, 1.2 μM each of primers YMV-FIP and YMV-BIP, 0.5 μM each of primers YMV-LF and YMV-LB, 1.2 mM each dNTP, 0.6 M betaine (Sigma-Aldrich, USA), 4 mM MgSO_4_, 8 U of *Bst* 2.0 polymerase (New England Biolabs, UK), 14 U of M-MLV reverse transcriptase (Promega, USA), and 1× isothermal amplification buffer (New England Biolabs, UK). The RT-LAMP reactions were incubated in a thermal cycler (GeneAmp PCR System 2700, Applied Biosystems, USA) or in a hot water bath set at 65 °C for 60 min and then heated at 80 °C for 5 min to terminate the reaction. RT-LAMP products were analyzed by electrophoresis in 1.5% (w/v) TAE agarose gels and visualized as described earlier. For the closed-tube (CT) format, RT-LAMP reaction conditions are essentially as described, but with an addition of 2 µl of a 1:20-diluted SYBR Green I dye (Sigma-Aldrich, USA) inside the cap of the reaction tube. At the end of the reaction, tubes were inverted to mix SYBR Green dye loaded in the cap with the reaction mixture, and the tubes were observed under visible light using a black background to observe color difference between the positive (green) and negative (orange) reactions. The same reaction tubes were also observed using SYBR Green detection settings under UV-light in a Gel Doc™ EZ Imager.

**Table 1:** RT-LAMP primers designed in this study for the detection of yam mosaic virus (YMV)

Primer name	Position^a^	Sequence (5’→3’)	Length	Orientation^d^
YMV-F3	609-626	GACAATGATGGACGGTGC	18	F
YMV-B3	799-819	GAAGTCAAACGCATATCTAGC	21	R
YMV-FIP (F1c+F2)^b^	F1c: 679-705 F2: 631-647	ACTGAAATGCATCATTATCTGACGAA- GCAAGTGGAATACCCATT	44	R F
YMV-BIP (B1c+B2)^b^	B1c: 715-741 B2: 777-795	GAAGCATACATTGAATTGCGGAACTCAA- TGAGTAATCCCTCAAGTTG	47	F R
YMV-LF^c^	650-674	GGTTTGGCATTTTCTATGATCGGTT	25	R
YMV-LB^c^	757-776	CCCCGATACGGTATTCAGCG	20	F

^a^Alignment position of the primers with the reference YMV sequence (GenBank accession no. AJ244066)

^b^The forward (YMV-FIP) and reverse (YMV-BIP) internal primers consist of two fragments in forward and reverse orientation. The joining site is indicated by a hyphen

^c^Loop primers

^d^F, forward orientation; R, reverse orientation

Experiments were conducted to detect YMV using a rapid virus release protocol without tissue homogenization as described by Thomson and Dietzgen [16], using alkaline PEG buffer (6% polyethylene glycol (PEG)-200 in 20 mM NaOH in sterile distilled water) [3]. In this case, yam leaf or tuber tissue (about 50 to 100 mg) was placed in a 1.5-ml microfuge tube containing 0.5 ml of alkaline PEG buffer, and the tubes were incubated at room temperature for 10 min with occasional shaking. Two µl of the sample was used as a template for YMV detection by CT-RT-LAMP with SYBR Green visualization.

Experiments to determine the detection sensitivity of CT-RT-LAMP and RT-PCR were conducted with 10x serial dilutions of total RNA (100 ng/µl stock) obtained from YMV-infected yam leaves in total RNA extracted from uninfected yam leaf up to a 10^−6^ dilution, and 2 µl from each dilution was used as a template for testing in duplicate. The assay sensitivity to detect YMV in bulk samples comprising 10 yam leaves per bulk was determined by including one leaf from a YMV-infected plant (variety TDr Danacha or TDr 09/00058) with nine leaves from healthy yam. Leaf discs from bulk samples were excised using a 1-cm-diameter cork borer and used for total RNA extraction or by soaking in alkaline PEG reagent as described earlier. Tenfold serial dilutions of total RNA (100 ng/µl stock) or extract in alkaline PEG reagent from bulk samples were performed, and 2 µl was used as template for CT-RT-LAMP and RT-LAMP. Conditions for CT-RT-LAMP and RT-PCR were as described above. Both agarose gel electrophoresis and visual detection with SYBR Green were used to verify the YMV detection limit of RT-LAMP, whereas the RT-PCR products were analyzed in agarose gels as described above. The performance of CT-RT-LAMP and RT-PCR in detecting YMV in over 50 field-collected *D. rotundata* and *D. alata* leaf and tuber samples was compared by analyzing total RNA (2 µl of 100 ng/µl stock).

The RT-PCR in YMV-positive samples resulted in amplification of the expected 320-bp product (Fig. 1E). The six RT-LAMP primers designed recognize eight binding sites in the YMV CP region. This includes two outer primers, YMV-F3 and YMV-B3, in the sense and antisense orientation, respectively; two internal primers, YMV-FIP and YMV-BIP, each containing two separate binding sites, referred to as F2 and F1C and B2 and B2C, in opposite orientations; and two loop primers, YMV-LF and YMV-LB (Table 1, Supplementary Fig. 1). To test the fragment length of the CP sequence targeted for LAMP detection, conventional RT-PCR assay was performed with the outer primer pair YMV-F3 and YMV-B3, keeping the RT-PCR reaction conditions similar to those used for YMV-F and YMV-R. This resulted in amplification of the expected 211-bp product (data not shown). The 211-bp RT-PCR product was sequenced in both orientations, and comparison of the resultingsequence (GenBank accession no. MF521884) by BLAST search revealed 97 to 100% sequence identity with YMV sequences available in the GenBank database, confirming the specificity of the primers for the virus genome. The six RT-LAMP primers designed in this study successfully detected YMV using a single incubation temperature of 65 °C for 60 min. Ladder-like patterns of amplified products in YMV-positive samples were detected in agarose gels (Fig. 1A). The specificity of the loop primers (YMV-LF and YMV-LB) in the RT-LAMP assay was evaluated by comparing assays with and without the addition of these primers to the reaction mixture (Fig. 1C and D). Use of loop primers at concentrations above 0.5 µM resulted in false-negative results, as some YMV-positive samples were not detected (Supplementary Fig. 2). The optimal primer conditions for the RT-LAMP were determined by testing different concentrations, and a ratio of inner to outer to loop primers of 7.5:1:3.125 was found to be optimal. The inclusion of loop primers, primer concentration, and relative ratio to each other had a crucial influence on the specificity and consistent amplification of the target region in YMV-positive samples.

**Fig. 1:**
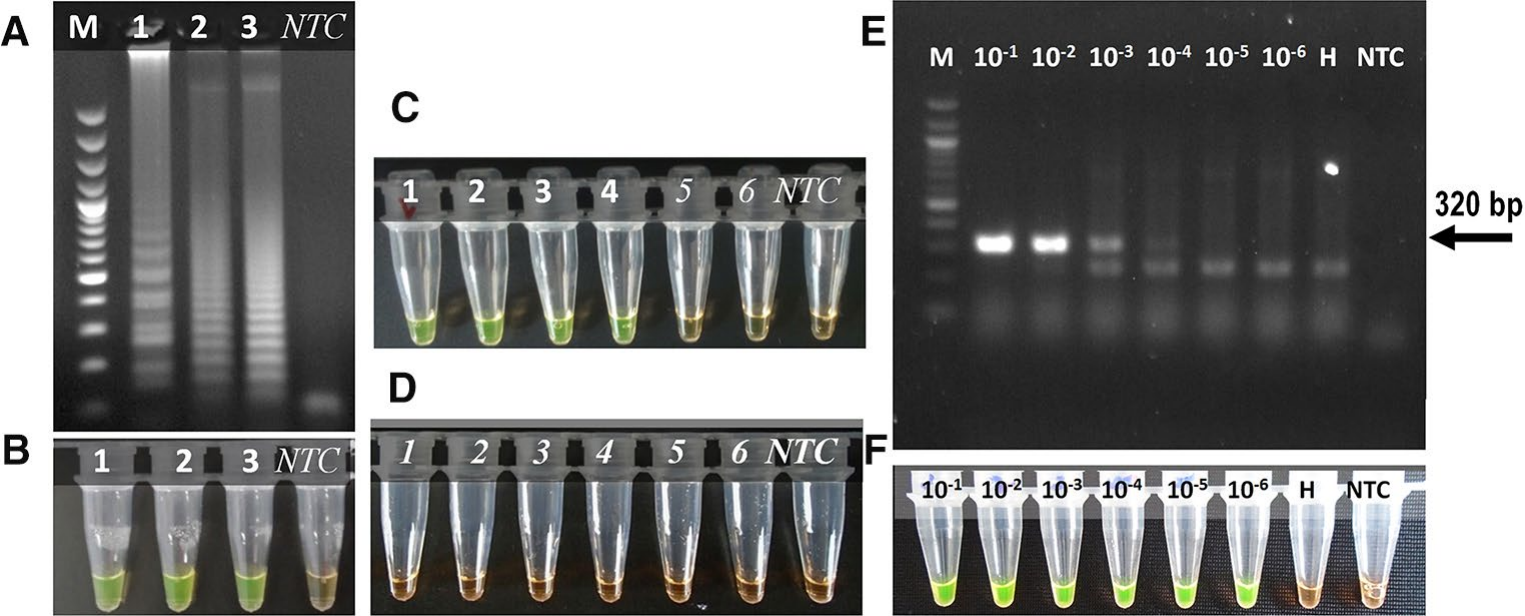
CT-RT-LAMP assays for YMV detection in yam. Amplification products were visualised by agarose gel electrophoresis (A) and with SYBR Green (B). Samples in lanes 1, 2 and 3 are YMV-infected samples. B, non-template control; M, 100-bp DNA ladder. (C) CT-RT-LAMP with loop primers. (D) CT-RT-LAMP, without loop primers. Samples in lanes 1 to 4: YMV-infected samples, 5 – 6, healthy yam samples. **(**E and F**)** Comparison between RT-PCR (E) and CT-RT-LAMP (F) for detecting YMV in infected yam leaf sample. Tenfold dilutions of total RNA from 100 ng/µl were tested. RT-PCR products were analyzed by agarose gel electrophoresis, and visual detection of RT-LAMP was done using SYBR Green dye. M, 100-bp DNA ladder; H, healthy yam RNA sample; NTC, no-template control

In CT format using SYBR Green dye for chromogenic detection, samples with positive amplification appeared green, and the YMV-negative samples and water control appeared orange under visual light using a black background (Fig. 2A). Bright green fluorescence was observed in YMV-positive samples, whereas negative samples were colourless when observed under UV light (SYBR Green setting) in a Gel Doc™ EZ Imager (Fig. 2B). The scoring of CT-RT-LAMP results as positive or negative was identical under visible light or UV-light (Fig. 2), indicating the suitability of visible scoring as simple alternative to UV-light-source-dependent detection of CT-RT-LAMP results.

**Fig. 2:**
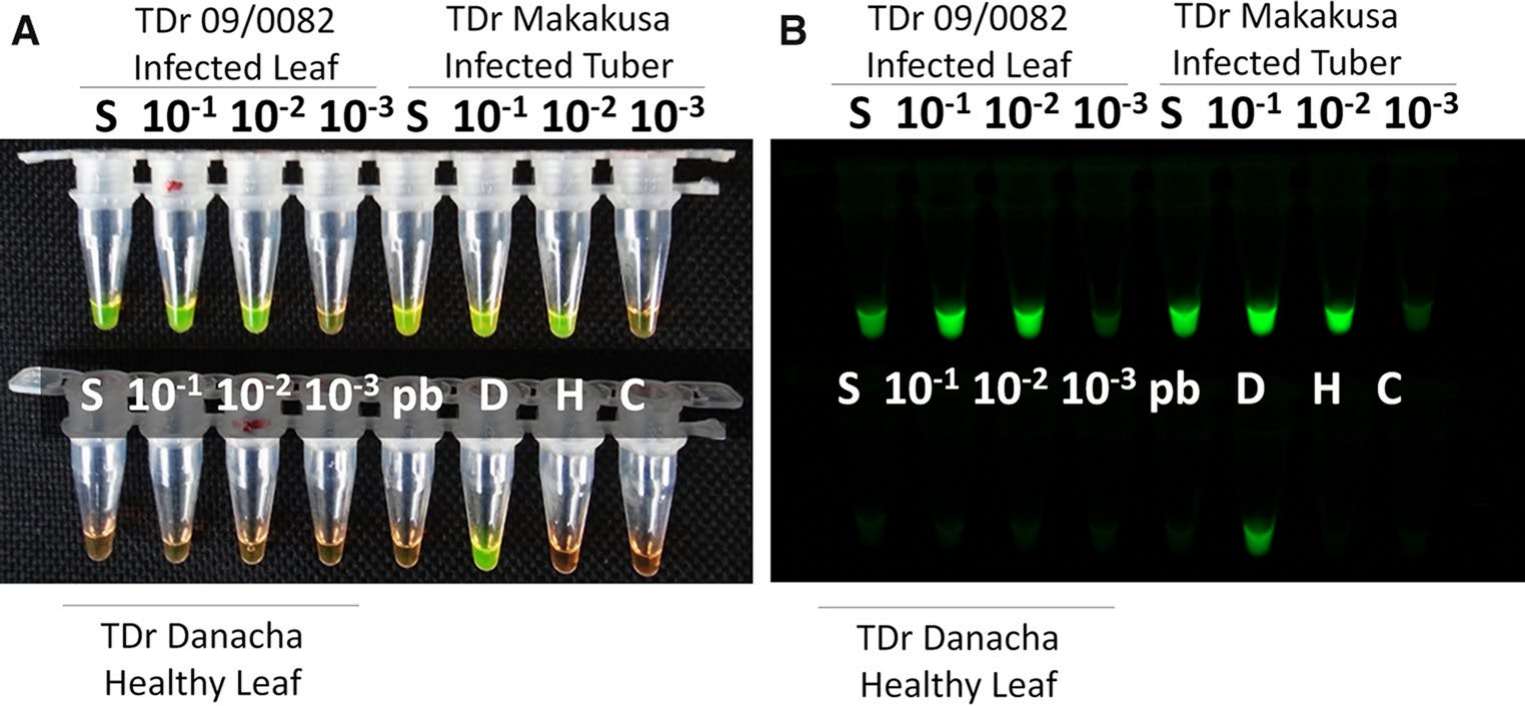
YMV detection in leaf and tuber sap samples by CT-RT-LAMP. Sap was extracted from 100 mg of tissue in alkaline PEG buffer (1:20 dilution), and 2 µl was used as template for virus detection. The genotype number of the yam, template dilution, and infection status are indicated. Pb, alkaline PEG buffer control; D, YMV positive control; H, healthy yam control; B, no-template control; S, undiluted stock

In experiments to determine the sensitivity of detection, the CT-RT-LAMP detected YMV at dilutions up to 10^−5^ to 10^−6^ using total RNA extracted by the CTAB or alkaline PEG method, whereas positive amplification was observed in the RT-PCR at dilutions up to 10^−3^ to 10^−4^, indicating that the RT-LAMP is at least 100 times more sensitive than RT-PCR (Fig. 1E and F). Assays conducted using samples soaked in alkaline PEG reagent resulted in successful detection of YMV up to 10^−3^ (v/v) dilution in both leaf (TDr 09/0082) and tuber (TDr Makakusa) samples (Fig. 2). Experiments to detect YMV in total RNA extracts of bulk samples (1:10 infected to healthy leaf sample ratio) resulted in virus detection up to a dilution of 10^−4^ in CT-RT-LAMP and up to a dilution of 10^−3^ in RT-PCR in two yam varieties tested (TDr Danacha and TDr 09/00058) (Supplementary Fig. 3A). However, YMV detection at a dilution of 10^−1^ consistently gave negative results in RT-PCR (lane 1, Supplementary Fig. 3A), but amplification was observed in CT-RT-LAMP (tube 1, Supplementary Fig. 3C). We suspect that a high template concentration may have an inhibitory effect on RT-PCR but not on RT-LAMP. Similar experiments to detect YMV in bulk samples using a sample soaked in alkaline PEG reagent as template in RT-PCR resulted in consistent detection of YMV at a 10^−2^ (v/v) dilution of total RNA (Supplementary Fig. 3B), but in CT-RT-LAMP, detection was achieved in samples diluted up to 10^−4^ to 10^−6^ (Supplementary Fig. 3D) in two yam varieties tested (TDr Danacha and TDr 09/00058). These experiments confirmed that CT-RT-LAMP offers relatively high sensitivity for YMV detection, and the results were consistent with those obtained by RT-PCR using either total RNA or sample soaked in alkaline PEG reagent. For routine testing of bulk samples consisting of 10 samples, a 10^−2^ dilution is advised.

Total RNA extracted from 25 leaf and tuber samples collected from yams grown in the screenhouse and field were tested with CT-RT-LAMP and RT-PCR (Supplementary Table 1). Twelve samples tested positive for YMV in both assays, and *D. alata* infected with yam mild mosaic virus (YMMV, genus *Potyvirus*) used as a nonspecific control, healthy yams, and the non-template control tested negative as expected. Samples taken from the head, middle, and bottom portions of yam tubers tested positive for YMV in CT-RT-PCR and RT-PCR (Supplementary Fig. 4), except for one sample which tested positive in CT-RT-LAMP and negative in RT-PCR (lane 18, Supplementary Fig. 4).

The CT-RT-LAMP assay is suitable for the detection of YMV in both leaf and tuber tissues without the need for a separate RNA extraction step and permits visual inspection of results without the need for expensive detectors. This has a substantial impact on the practicability of the assay, not only reducing the cost of consumables associated with agarose gel electrophoresis but also potentially reducing handling time and risks of carryover contamination. Because of these features, we are hopeful that CT-RT-LAMP will be adopted for routine detection of YMV, especially to verify the infection status of planting material in West Africa, where active efforts are ongoing to invigorate production of virus-free seed yam necessary for sustainable seed systems [8].

## Electronic supplementary material

Below is the link to the electronic supplementary material.

Supplementary material 1 (JPEG 1468 kb) Supplementary Fig. 1 Relative position of LAMP primers on the YMV reference sequence (NCBI GenBank accession no. AJ244066). Primer sequences are highlighted, and the direction of amplification is indicated by arrows (continuous line for forward [sense]-direction primer and discontinuous line for reverse [complementary] primer)

Supplementary material 2 (JPEG 809 kb) Supplementary Fig. 2 Evaluation of the effect of different concentrations of loop primers on RT-LAMP sensitivity. A, 1 µM; B, 0.8 µM; C, 0.5 µM; D, 1.2 µM. RT-LAMP amplification products were resolved in 1.5% agarose gels. Samples 1 and 15 are YMV positive, and dilutions of 1:20 and 1:100 of total RNA at an initial concentration of 100 ng/µl were used. NTC, non-template control; M, 100-bp DNA ladder

Supplementary material 3 (JPEG 1672 kb) Supplementary Fig. 3 Comparative detection of YMV in bulk samples made with 1:9 YMV-infected to healthy yam leaves using RT-PCR (A and B) and CT-RT-LAMP (C and D) using total RNA extracts (A and C) and sample soaked in alkaline PEG reagent (B and D). The sources of the YMV-infected cultivar, TDr Danacha (Dan) and TDr 09/0058 (58), and the weight of the sample tissue used for extraction, 1:10 (w/v) and 1:20 (w/v), are indicated. Extracted samples (total RNA or sample soaked in alkaline PEG reagent) were diluted 10^−1^ to 10^−6^ (lanes or tubes 1 to 6). D1, D2, and D3 are YMV positive controls. H, healthy yam; B, no-template control; M, 100-bp DNA ladder. RT-PCR products (A and C) were resolved in 1.5% TAE agarose gels stained with EZ Blue dye and visualized under UV light, whereas CT-RT-LAMP (B and D) products were visualized using SYBR Green dye and visualized under UV light. The sample loading order was identical in agarose gels and CT-RT-LAMP

Supplementary material 4 (JPEG 1592 kb) Supplementary Fig. 4 CT-RT-LAMP for the detection of YMV in seed yam tubers of cultivar TDr Danacha. Chromogenic detection under visible light (A), UV light (B), and corresponding detection by RT-PCR (C). Details of the samples tested are given in the Table 1. Numbers on the tubes or gels correspond to the sample number given in the Table. ‘+’, positive RT-PCR amplification; ‘-‘, no amplification in RT-PCR. Reaction conditions for CT-RT-LAMP and RT-LAMP are essentially as described in the manuscript, except that the oligonucleotide primers used for RT-PCR were YMV-F3x and YMV-B3x

Supplementary material 5 (DOCX 15 kb)
