# Supplementary figures and images for: Chromogenic detection of yam mosaic virus by closed-tube reverse transcription loop-mediated isothermal amplification (CT-RT-LAMP)
Source: Arch Virol. 2018 Jan 8;163(4):1057–61. doi: 10.1007/s00705-018-3706-0 (PMC5854734; doi:10.1007/s00705-018-3706-0)

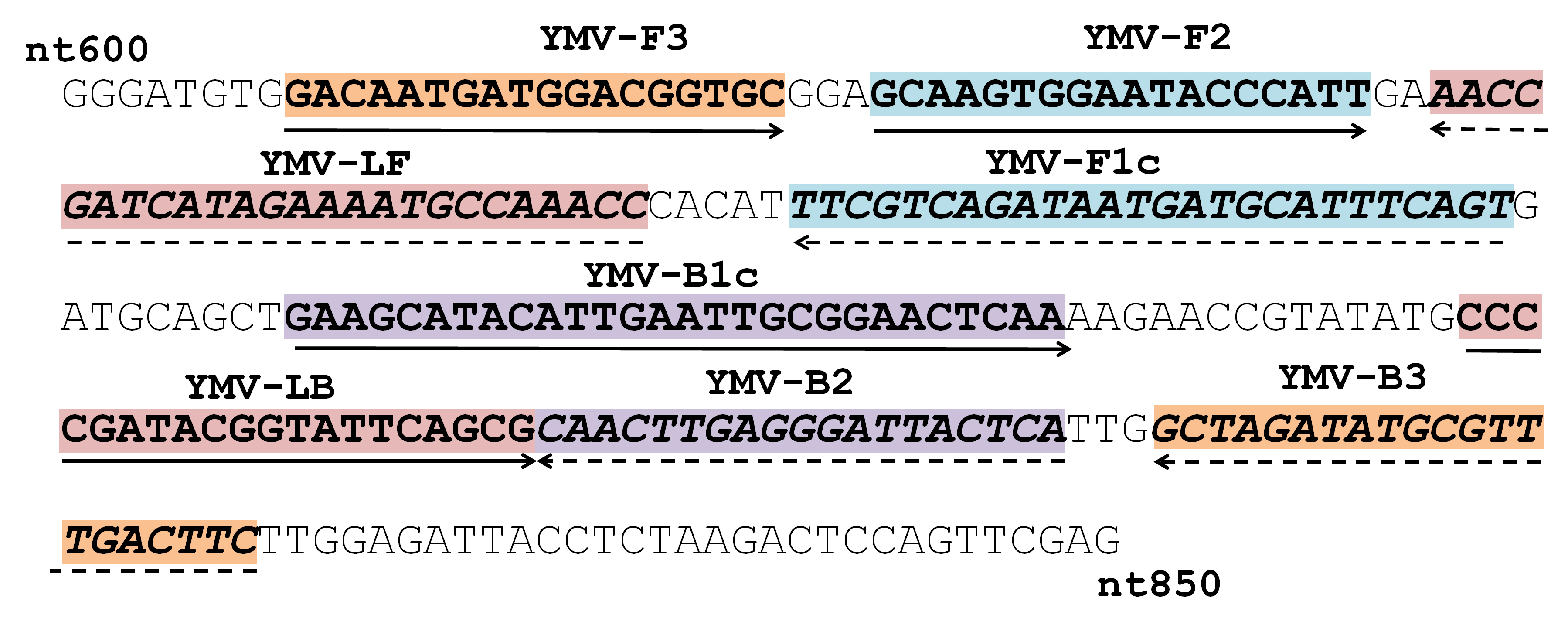

Supplement: Supplementary file 1 — Supplementary material 1 (JPEG 1468 kb) Supplementary Fig. 1 Relative position of LAMP primers on the YMV reference sequence (NCBI GenBank accession no. AJ244066). Primer sequences are highlighted, and the direction of amplification is indicated by arrows (continuous line for forward [sense]-direction primer and discontinuous line for reverse [complementary] primer) [file 705_2018_3706_MOESM1_ESM.jpg]

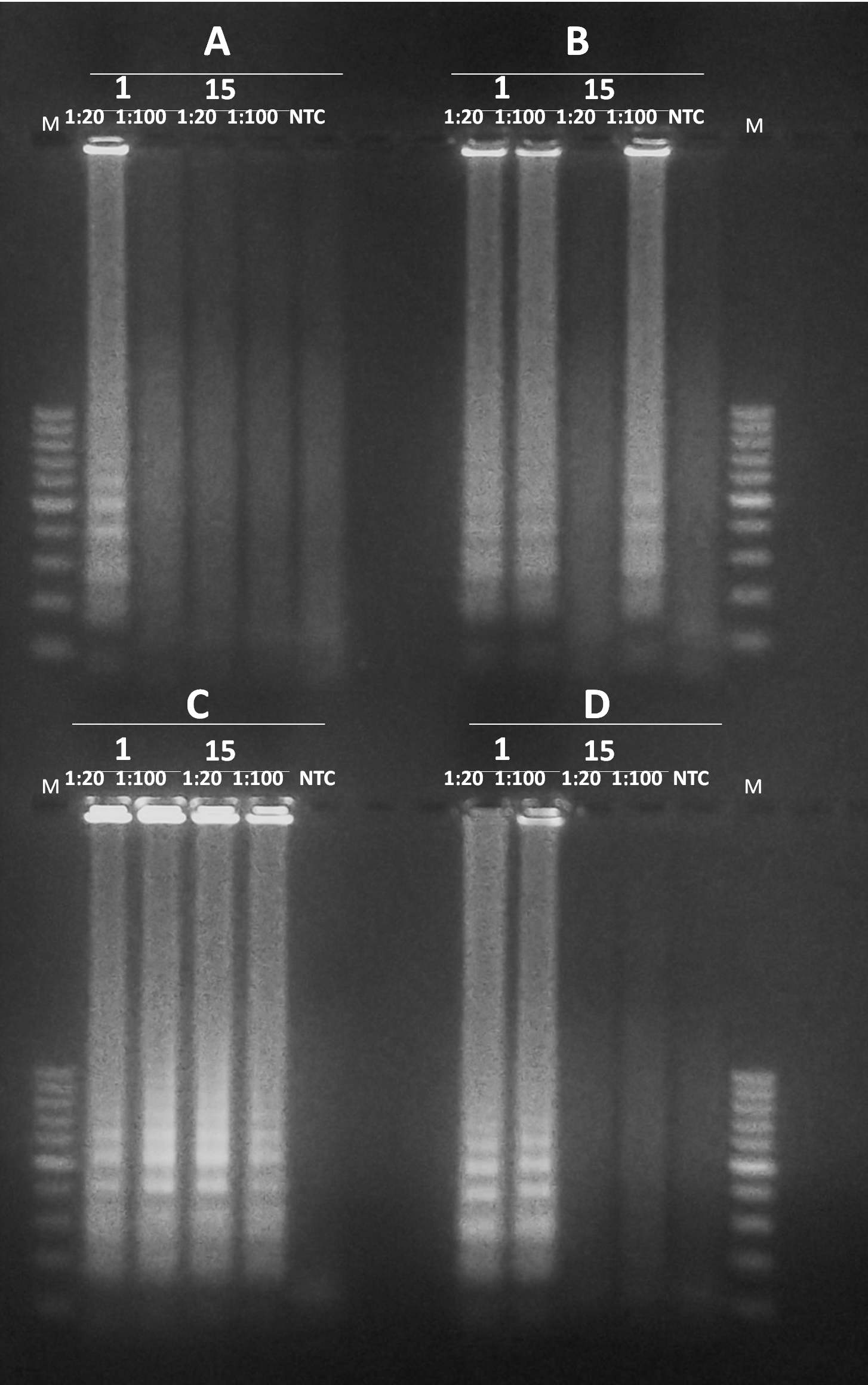

Supplement: Supplementary file 2 — Supplementary material 2 (JPEG 809 kb) Supplementary Fig. 2 Evaluation of the effect of different concentrations of loop primers on RT-LAMP sensitivity. A, 1 µM; B, 0.8 µM; C, 0.5 µM; D, 1.2 µM. RT-LAMP amplification products were resolved in 1.5% agarose gels. Samples 1 and 15 are YMV positive, and dilutions of 1:20 and 1:100 of total RNA at an initial concentration of 100 ng/µl were used. NTC, non-template control; M, 100-bp DNA ladder [file 705_2018_3706_MOESM2_ESM.jpg]

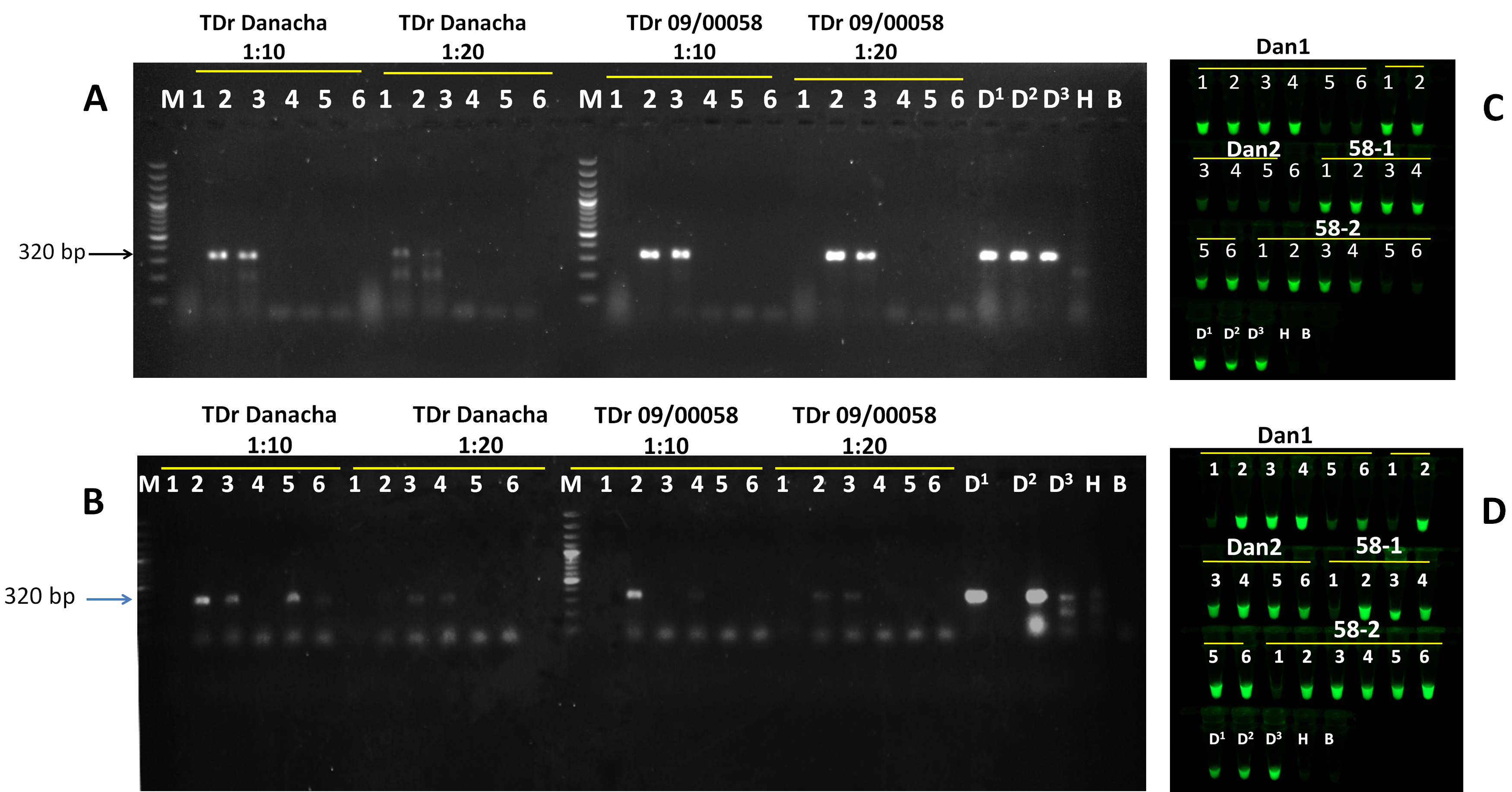

Supplement: Supplementary file 3 — Supplementary material 3 (JPEG 1672 kb) Supplementary Fig. 3 Comparative detection of YMV in bulk samples made with 1:9 YMV-infected to healthy yam leaves using RT-PCR (A and B) and CT-RT-LAMP (C and D) using total RNA extracts (A and C) and sample soaked in alkaline PEG reagent (B and D). The sources of the YMV-infected cultivar, TDr Danacha (Dan) and TDr 09/0058 (58), and the weight of the sample tissue used for extraction, 1:10 (w/v) and 1:20 (w/v), are indicated. Extracted samples (total RNA or sample soaked in alkaline PEG reagent) were diluted 10−1 to 10−6 (lanes or tubes 1 to 6). D1, D2, and D3 are YMV positive controls. H, healthy yam; B, no-template control; M, 100-bp DNA ladder. RT-PCR products (A and C) were resolved in 1.5% TAE agarose gels stained with EZ Blue dye and visualized under UV light, whereas CT-RT-LAMP (B and D) products were visualized using SYBR Green dye and visualized under UV light. The sample loading order was identical in agarose gels and CT-RT-LAMP [file 705_2018_3706_MOESM3_ESM.jpg]

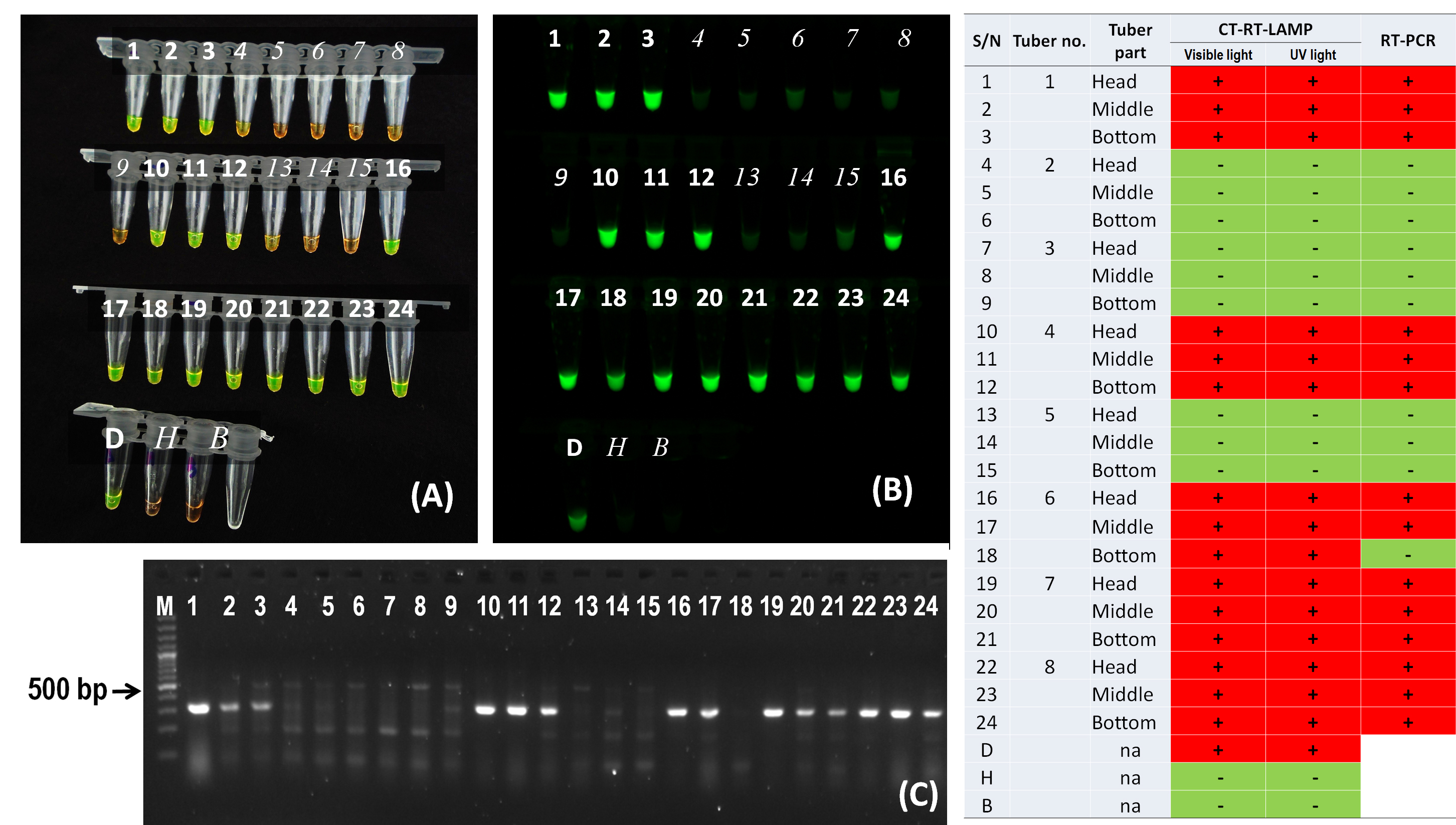

Supplement: Supplementary file 4 — Supplementary material 4 (JPEG 1592 kb) Supplementary Fig. 4 CT-RT-LAMP for the detection of YMV in seed yam tubers of cultivar TDr Danacha. Chromogenic detection under visible light (A), UV light (B), and corresponding detection by RT-PCR (C). Details of the samples tested are given in the Table 1. Numbers on the tubes or gels correspond to the sample number given in the Table. ‘+’, positive RT-PCR amplification; ‘-‘, no amplification in RT-PCR. Reaction conditions for CT-RT-LAMP and RT-LAMP are essentially as described in the manuscript, except that the oligonucleotide primers used for RT-PCR were YMV-F3x and YMV-B3x [file 705_2018_3706_MOESM4_ESM.jpg]
