# Supplementary material for: Chromogenic detection of yam mosaic virus by closed-tube reverse transcription loop-mediated isothermal amplification (CT-RT-LAMP)
Source: Arch Virol. 2018 Jan 8;163(4):1057–61. doi: 10.1007/s00705-018-3706-0 (PMC5854734; doi:10.1007/s00705-018-3706-0)
Supplement: Supplementary file 5 — Supplementary material 5 (DOCX 15 kb) [file 705_2018_3706_MOESM5_ESM.docx]

Supplementary Table 1. Comparison on YMV detection in leaf and tubers samples sourced from screen house and fields in Nigeria by RT-PCR and CT-RT-LAMP. Total RNA (100 ng/µl) extracted from 100 mg leaf sample or a piece from the head portion of yam tuber was used as template

| Sample ID | CT-RT-LAMP | RT-PCR |
| --- | --- | --- |
| TDr Makakusa 3M^1^ | + | + |
| TDr Makakusa 7H^1^ | + | + |
| TDr Gbangu 15^1^ | + | + |
| TDr Amola^1^ | + | + |
| TDr Danacha^1^ | + | + |
| TDr 97/ 00840^1^ | + | + |
| TDr 07/ 00033^1^ | + | + |
| TDr 95/19177^1^ | + | + |
| TDr 07/00873^1^ | + | + |
| TDr 03/00180^1^ | + | + |
| TDr Ogini M3^2^ | - | - |
| TDr Ogini H41^2^ | - | - |
| TDr Aloshi (H1)B1^2^ | - | - |
| TDr Aloshi B1 21M^2^ | - | - |
| TDa 92-2 (YMMV)^3^ | - | - |
| TDa 11/00316 (YMMV)^3^ | - | - |
| TDa 11/00203 (YMMV)^3^ | - | - |
| TDa 297 (YMMV)^3^ | - | - |
| TDa 11/00300 (YMMV)^3^ | - | - |
| TDa 11/00193 (YMMV)^3^ | - | - |
| TDa 11/00302 (YMMV)^4^ | + | + |
| TDa 11/00204 (YMMV)^4^ | + | + |
| TDr 09/00002^1^ | + | + |
| TDa 11/00203 (YMMV)^3^ | - | - |
| TDr Aloshi^2^ | - | - |
| TDr = *Dioscorea rotundata*; TDa = *D. alata*  YMV = Yam mosaic virus, YMMV = Yam mild mosaic virus  Total RNA from YMV infected^1^ and healthy^2^ yam;  *D. alata* infected with YMMV^3^ ; *D. alata* infected with YMV + YMMV^4^  + = YMV positive; - = YMV negative | | |
